# Nomogram models for stratified prediction of axillary lymph node metastasis in breast cancer patients (cN0)

**DOI:** 10.3389/fendo.2022.967062

**Published:** 2022-08-30

**Authors:** Xin Gao, Wenpei Luo, Lingyun He, Lu Yang

**Affiliations:** ^1^ Department of Breast and Thyroid Surgery, Second Affiliated Hospital of Chongqing Medical University, Chongqing, China; ^2^ Scientific Research and Education Section, Chongqing Health Center for Women and Children, Chongqing, China

**Keywords:** breast cancer, axillary lymph node metastasis, predictor, nomogram model, stratified prediction

## Abstract

**Objectives:**

To determine the predictors of axillary lymph node metastasis (ALNM), two nomogram models were constructed to accurately predict the status of axillary lymph nodes (ALNs), mainly high nodal tumour burden (HNTB, > 2 positive lymph nodes), low nodal tumour burden (LNTB, 1-2 positive lymph nodes) and negative ALNM (N0). Accordingly, more appropriate treatment strategies for breast cancer patients without clinical ALNM (cN0) could be selected.

**Methods:**

From 2010 to 2015, a total of 6314 patients with invasive breast cancer (cN0) were diagnosed in the Surveillance, Epidemiology, and End Results (SEER) database and randomly assigned to the training and internal validation groups at a ratio of 3:1. As the external validation group, data from 503 breast cancer patients (cN0) who underwent axillary lymph node dissection (ALND) at the Second Affiliated Hospital of Chongqing Medical University between January 2011 and December 2020 were collected. The predictive factors determined by univariate and multivariate logistic regression analyses were used to construct the nomograms. Receiver operating characteristic (ROC) curves and calibration plots were used to assess the prediction models’ discrimination and calibration.

**Results:**

Univariate analysis and multivariate logistic regression analyses showed that tumour size, primary site, molecular subtype and grade were independent predictors of both ALNM and HNTB. Moreover, histologic type and age were independent predictors of ALNM and HNTB, respectively. Integrating these independent predictors, two nomograms were successfully developed to accurately predict the status of ALN. For nomogram 1 (prediction of ALNM), the areas under the receiver operating characteristic (ROC) curve in the training, internal validation and external validation groups were 0.715, 0.688 and 0.876, respectively. For nomogram 2 (prediction of HNTB), the areas under the ROC curve in the training, internal validation and external validation groups were 0.842, 0.823 and 0.862. The above results showed a satisfactory performance.

**Conclusion:**

We established two nomogram models to predict the status of ALNs (N0, 1-2 positive ALNs or >2 positive ALNs) for breast cancer patients (cN0). They were well verified in further internal and external groups. The nomograms can help doctors make more accurate treatment plans, and avoid unnecessary surgical trauma.

## Introduction

Breast cancer remains one of the most lethal diseases that threatens women’s health, and its incidence has ranked first among all tumours in 2020 (1, 2). Although breast cancer relies on systemic treatment options, the primary means for most early-stage breast cancer patients remains surgical intervention (3). Minimally invasive breast surgery, such as breast-conserving surgery and sentinel lymph node biopsy (SLNB), has become more common (4, 5). Axillary lymph node metastasis (ALNM) is one of the most crucial factors affecting locoregional recurrence (LRR) and overall survival (6). The 5-year overall survival rate of breast cancer patients without ALNM is as high as 99%, but it decreases to 85.8% for ALNM patients (7). Therefore, accurate assessment of axillary lymph node status is essential for optimal treatment of breast cancer. Although conventional ultrasound and MRI provide great help for the preoperative evaluation of axillary lymph node status, there are still many uncertainties, especially for small lymph nodes. Valente et al. and Hwang et al. found that the false negative rates (FNR) of predicting axillary lymph node positivity by axillary ultrasound were as high as 16.7% and 22.9%, respectively (8, 9).

Given that axillary lymph node dissection (ALND) can lead to a variety of surgical complications, including postoperative upper limb oedema, pain, numbness, and dysfunction (10, 11), the application of SLNB reduces the complications of ALND to a large extent without compromising staging, survival, or local-regional recurrence (12, 13). Although SLNB is less invasive than ALND, approximately 25% of patients still have short-term complications such as lymphedema and pain, which leads to a significant decline in quality of life (14, 15). At present, the method recommended in the guidelines is still the combined method (nuclide tracer and dye) (16). However, dye-related life-threatening anaphylactic reactions occur in approximately 0.25% to 0.5% of patients (17), and the nuclide tracer causes radioactive exposure to both patients and surgeons.

As a consensus, no further axillary surgery is required in cases of negative sentinel lymph nodes (SLNs) (18). However, whether to perform ALND is still controversial in cases of low nodal tumour burden (LNTB, 1-2 positive lymph nodes). The International Breast Cancer Study Group (IBCSG) 23–01 trials concluded that ALND should be avoided for patients with one or more micro-metastatic (≤ 2 mm) SLNs and tumours ≤ 5 cm; as long as patients receive traditional whole-breast radiotherapy and systemic adjuvant therapy, there is no adverse effect on survival (19). The American College of Surgeons Oncology Group (ACOSOG) Z0011 trial demonstrated that patients (T1-2 and cN0) with 1-2 positive SLNs were exempt from ALND by breast-conserving surgery with subsequent radiotherapy and adjuvant therapy (20, 21). While SLNB is the current standard for axillary staging in breast cancer patients (cN0), SLN is negative in approximately 76% of patients (22, 23). Therefore, if accurate preoperative prediction of ALNs can be achieved, SLNB for lymph node-negative patients or SLNB + ALND for LNTB patients would become unnecessary. For high nodal tumour burden patients (HNTB, > 2 positive lymph nodes), it would be beneficial to the choice of further treatment, including neoadjuvant therapy and surgical procedure.

In summary, accurate prediction of ALN status is helpful for reducing unnecessary ALND and SLNB, which is beneficial for individualized and accurate treatment plans. Many studies have focused on the risk factors for ALNM in breast cancer patients (cN0). Zeng et al. found that grade, estrogen receptor (ER) status and human epidermal growth factor receptor 2 (Her-2) status were significantly correlated with ALNM (24). Hu et al. concluded that a significant association between young age, high BMI, high Ki67 and large tumour size was an independent predictor for ALNM (25). However, the accuracy of the above studies remains uncertain due to insufficient population size.

In this study, we used the Surveillance, Epidemiology, and End Results (SEER) database of the National Cancer Institute to identify the factors that affect ALNM and then constructed nomogram models to predict the probability of ALNM (metastases, LNTB and HNTB or not), with the ultimate goal of helping doctors make appropriate treatment plans preoperatively for patients with breast cancer (cN0).

## Material and methods

### Data source

The data we analyzed were extracted from two databases. First, training and internal validation groups on demographic characteristics and cancer incidence in the United States population were obtained from the SEER Program (https://seer.cancer.gov/). Second, the external validation group was from the Second Affiliated Hospital of Chongqing Medical University.

### Patient selection

In the SEER Program, we screened female patients diagnosed with invasive breast cancer (ICD-O-3 codes 8500, 8480/3, 8401/3, 8510/3, 8520/3, 8521/3, 8522/3-8524/3) between 2010 and 2015. Variables included age, race, laterality, primary site, molecular subtype, ER status, progesterone receptor (PR) status, Her-2 status, grade, tumour size, histologic type, total number of postoperative (ALND) lymph nodes and positive lymph nodes. Inclusion criteria included female invasive breast cancer, cN0, unilateral and single tumour. The exclusion criteria included clinical positive axillary lymph node and distant metastasis, multiple breast cancer lesions, neoadjuvant therapy, coexistence with other tumours, and incomplete medical data.

In the Second Affiliated Hospital of Chongqing Medical University, the data of patients with invasive breast cancer were collected between January 2011 and December 2020. The patients were enrolled in this study according to the inclusion and exclusion criteria above.

### Grouping

According to the National Comprehensive Cancer Network (NCCN) guidelines and the ACOSOG Z0011 trial (20, 21, 26), ALND is not routinely recommended for patients with 1-2 lymph node metastases for their low lymph node tumor burden. Hence, the patients enrolled in this study were divided into three groups: 1. N0 (without any axillary lymph node metastasis); 2. LNTB (1-2 axillary lymph node metastasis); 3. HNTB (> 2 axillary lymph node metastasis).

### Statistical analysis

After screening by inclusion and exclusion criteria, 6314 patients from the SEER database and 503 patients from the Second Affiliated Hospital of Chongqing Medical University were ultimately included in this study. The patients from the SEER database were randomized 3:1 to the training and internal validation groups for the construction and verification of the nomogram, respectively.

SPSS 26.0 software was used for analysis. In univariate analysis, the chi-square test was used for the comparison of categorical variables. Multivariate regression analysis was used to identify the independent predictors in patients. The rms package for R software (Version 4.1.0; https://www.r-project.org) was used to establish nomograms based on the significantly independent predictors. Then, two validation groups were used for internal and external verification. The receiver operating characteristic (ROC) curves of the prediction model and the verification groups were drawn, and the area under the curve (AUC) was calculated to evaluate the discrimination of the prediction models. Two calibration curves were plotted to assess the nomogram prediction ability *via* the comparison between predictive and actual ALN status. P < 0.05 was considered statistically significant.

## Results

### Patients’ characteristics

From 2010 to 2015, 6314 patients in the SEER database were included, and 503 patients in the Second Affiliated Hospital of Chongqing Medical University were included from January 2011 to December 2020. The SEER data were divided into two groups: the training group (n = 4751) and the internal validation group (n = 1563). Data from the Second Affiliated Hospital of Chongqing Medical University were used as the external validation group (n = 503). The specific clinicopathological features of the patients in the training and validation groups are summarized in **Table 1**. In the training group, ALNM was 49.1% (2332/4751), LNTB was 41.3% (1961/4751), and HNTB was 7.8% (371/4751). Approximately 47.0% (734/1563, including 38.9% LNTB and 8.1% HNTB) of patients were identified as having ALNM in the internal validation group, whereas 41.0% (206/503, including 22.3% LNTB and 18.7% HNTB) of patients were identified as having ALNM in the external validation group.

**Table 1:** Clinicopathological characteristics of patients with breast cancer in SEER Program and our center.

Variables	Subgroup	No. (%) of patients		
		Training group (n = 4751)	Internal group (n = 1563)	External group (n = 503)
Age (year)	20~39	95 (2.0)	42 (2.6)	51 (10.1)
	40~59	1718 (36.2)	581 (37.2)	283 (56.3)
	≥60	2938 (61.8)	940 (60.2)	169 (33.6)
Race	White	3847 (81.0)	1179 (75.4)	/
	Black	390 (8.2)	164 (10.4)	
	^a^Other	514 (10.8)	220 (14.2)	503 (100.0)
Tumor size (mm)	≤20	3265 (68.7)	1044 (66.8)	273 (54.3)
	20~50	1319 (27.8)	444 (28.4)	215 (42.7)
	>50	167 (3.5)	75 (4.8)	15 (3.0)
Primary site	Central	406 (8.5)	99 (6.5)	33 (6.6)
	Upper outer	2545 (53.6)	865 (55.3)	276 (54.9)
	Lower outer	574 (12.1)	176 (11.2)	58 (11.5)
	Upper inner	849 (17.9)	288 (18.4)	97 (19.3)
	Lower inner	377 (7.9)	135 (8.6)	39 (7.8)
Laterality	left	2395 (50.4)	752 (48.2)	279 (55.5)
	right	2356 (49.6)	811 (51.8)	224 (44.5)
Molecular subtype	Luminal A	4372 (92.0)	1415 (90.5)	277 (55.1)
	Luminal B	169 (3.6)	64 (4.0)	87 (17.3)
	TNBC	56 (1.2)	22 (1.5)	49 (9.7)
	HER2 enriched	154 (3.2)	62 (4.0)	90 (17.9)
ER	Positive	4530 (95.3)	1477 (94.5)	361 (71.8)
	Negative	221 (4.7)	86 (5.5)	142 (28.2)
PR	Positive	4163 (87.6)	1334 (85.4)	315 (62.6)
	Negative	588 (12.4)	229 (14.6)	188 (37.4)
HER2	Positive	225 (4.7)	86 (5.5)	105 (20.9)
	Negative	4526 (95.3)	1477 (94.5)	398 (79.1)
^b^Grade	I	1618 (34.0)	507 (32.5)	111 (22.1)
	II	2384 (50.2)	802 (51.3)	319 (63.4)
	III	749 (15.8)	254 (16.2)	73 (14.5)
Histologic type	IDC	3611 (76.1)	1124 (71.9)	355 (70.6)
	ILC	539 (11.3)	196 (12.6)	37 (7.4)
	IDC+ILC	424 (8.9)	120 (7.7)	43 (8.5)
	^c^Other	177 (3.7)	123 (7.8)	68 (13.5)
No. of positive ALNs	0	2419 (50.9)	829 (53.0)	297 (59.0)
	1~2	1961 (41.3)	607 (38.9)	112 (22.3)
	>2	371 (7.8)	127 (8.1)	94 (18.7)

a: the American Indian/Alaska Native and Asian/Pacific Islander;

b: Grade I: well-differentiated, Grade II: moderately differentiated, and Grade III: poorly differentiated;

c: mucinous carcinoma, medullary carcinoma, tubular carcinoma, and metaplastic carcinoma.

TNBC, triple-negative breast cancer; ER, estrogen receptor; PR, progesterone receptor; HER2, human epidermal growth factor receptor 2; IDC, invasive ductal carcinoma; ILC, invasive lobular carcinoma.

### Univariate analysis

Univariate analysis (**Table 2**) showed that age, race, tumour size, primary site, molecular subtype, histologic type, grade, ER status and PR status (p < 0.05) had statistical significance for ALNM in invasive breast cancer. However, race, Her-2 status and laterality were not statistically significant (P > 0. 05).

**Table 2:** Univariate analysis of ALNM in the training group.

Variables	Subgroup	No. (%) of patients			P
		LN - (n = 2419)	LN + (1-2) (n = 1961)	LN + (>2) (n = 371)	
Age (year)	20~39	43 (1.8)	40 (2.0)	12 (3.2)	<0.001
	40~59	916 (37.9)	709 (36.2)	93 (25.1)
	≥60	1460 (60.4)	1212 (61.8)	266 (71.7)
Race	White	1973 (81.6)	1576 (80.4)	298 (80.3)	0.04
	Black	171 (7.1)	181 (9.2)	38 (10.2)
	Other	275 (11.4)	204 (10.4)	35 (9.4)
Tumor size (mm)	≤20	2019 (83.5)	1178 (60.1)	68 (18.3)	<0.001
	20~50	377 (15.6)	714 (36.4)	228 (61.5)
	>50	23 (1.0)	69 (3.5)	75 (20.2)
Primary site	Central	146 (6.0)	198 (10.1)	62 (16.7)	<0.001
	Upper inner	541 (22.4)	274 (14.0)	34 (9.2)
	Lower inner	214 (8.8)	142 (7.2)	21 (5.7)
	Upper outer	1253 (51.8)	1082 (55.2)	210 (56.6)
	Lower outer	265 (11.0)	265 (13.5)	44 (11.9)
Laterality	left	1229 (50.8)	970 (49.5)	196 (52.8)	0.423
	right	1190 (49.2)	991 (50.5)	175 (47.2)
Molecular subtype	Luminal A	2250 (93.0)	1821 (92.9)	301 (81.1)	<0.001
	Luminal B	63 (2.6)	78 (4.0)	28 (7.5)
	TNBC	21 (0.9)	17 (0.9)	18 (4.9)
	HER2 enriched	85 (3.5)	45 (2.3)	24 (6.5)
ER	Positive	2307 (95.4)	1895 (96.6)	328 (88.4)	<0.001
	Negative	112 (4.6)	66 (3.4)	43 (11.6)
PR	Positive	2122 (87.7)	1755 (89.5)	286 (77.1)	<0.001
	Negative	297 (12.3)	206 (10.5)	85 (22.9)
HER2	Positive	84 (3.5)	95 (4.8)	46 (12.4)	0.05
	Negative	2335 (96.5)	1866 (95.2)	325 (87.6)
Grade	I	1022 (42.2)	548 (27.9)	48 (12.9)	<0.001
	II	1130 (46.7)	1074 (54.8)	180 (48.5)
	III	267 (11.0)	339 (17.3)	143 (38.5)
Histologic type	IDC	1852 (76.6)	1493 (76.1)	266 (71.7)	<0.001
	ILC	251 (10.4)	225 (11.5)	63 (17.0)
	IDC+ILC	192 (7.9)	200 (10.2)	32 (8.6)
	Other	124 (5.1)	43 (2.2)	10 (2.7)

LN−, disease-free axillae; LN+, any nodal metastasis; LN+ (1–2), 1 or 2 nodal metastasis; LN+ (>2), more than 2 nodal metastases.

### Multivariate logistic regression analysis

The above statistically significant factors were included in multivariate logistic regression analysis (**Table 3**). The results showed that tumour size (20-50 mm, odds ratio (OR) = 3.682, 95% CI: 3.181-4.267; >50 mm, OR = 9.725, 95% CI: 6.240-15.827; p < 0.001), primary site (central, p < 0.001), molecular subtype (Luminal B, OR = 1.127, 95% CI: 0.794-1.609; p < 0.001), grade (II, OR = 1.584, 95% CI: 1.380-1.819; III, OR = 2.311, 95% CI: 1.865-2.868; p<0.001) and histologic type [invasive ductal carcinoma (IDC) + invasive lobular carcinoma (ILC), OR = 1.178, 95% CI: 0.947-1.467; p < 0.001] were independent predictors of ALNM; and age (20-39, p = 0.006), tumour size (20 mm-50 mm, OR = 7.052, 95% CI: 5.628-10.000; > 50 mm, OR = 29.385, 95% CI: 19.613-44.027; p < 0.001), primary site (central, p < 0.001), molecular subtype (Luminal B, OR = 1.328, 95% CI: 0.808-2.122; triple-negative breast cancer (TNBC), OR = 8.097, 95% CI: 1.027-178.441; Her-2 enriched, OR =2.528, 95% CI: 0.337-54.416; p = 0.004) and grade (II, OR = 1.755, 95% CI: 1.243-2.478; III, OR = 3.468, 95% CI: 2.355-5.107; p < 0.001) were independent predictors of HNTB.

**Table 3:** Multivariate logistic regression analysis of ALNM in the training group.

Variables	Subgroup	LN− vs LN+		LN− and LN+ (1–2) vs LN+ (>2)	
		OR (95% CI)	P	OR (95% CI)	P
Age (year)	20~39	Reference	0.399	Reference	0.006
	40~59	1.007 (0.635-1.591)		0.581 (0.541-1.220)	
	≥60	1.099 (0.697-1.729)		0.883 (0.499-1.820)	
Race	White	Reference	0.104	Reference	0.549
	Black	1.181 (0.939-1.487)		0.883 (0.582-1.310)	
	Other	0.862 (0.704-1.055)		0.818 (0.541-1.205)	
Tumor size (mm)	≤20	Reference	<0.001	Reference	<0.001
	20~50	3.682 (3.181-4.267)		7.052 (5.628-10.000)	
	>50	9.725 (6.240-15.827)		29.385 (19.613-44.027)	
Primary site	Central	Reference	<0.001	Reference	<0.001
	Upper inner	0.362 (0.277-0.471)		0.323 (0.198-0.520)	
	Lower inner	0.487 (0.357-0.663)		0.468 (0.260-0.815)	
	Upper outer	0.653 (0.516-0.825)		0.660 (0.468-0.941)	
	Lower outer	0.766 (0.577-1.014)		0.635 (0.399-1.005)	
Molecular subtype	Luminal A	Reference	<0.001	Reference	0.004
	Luminal B	1.127 (0.794-1.609)		1.328 (0.808-2.122)	
	TNBC	0.894 (0.494-1.650)		8.097 (1.027-178.441)	
	HER2 enriched	0.442 (0.302-0.644)		2.528 (0.337-54.416)	
ER	Positive	2.909 (0.779-11.368)	0.110	0.337 (0.035-3.249)	0.347
	Negative	Reference		Reference	
PR	Positive	1.145 (0.906-1.449)	0.255	1.332 (0.901-1.969)	0.151
	Negative	Reference		Reference	
Grade	I	Reference		Reference	
	II	1.584 (1.380-1.819)	<0.001	1.755 (1.243-2.478)	<0.001
	III	2.311 (1.865-2.868)		3.468 (2.355-5.107)	
Histologic type	IDC	Reference	<0.001	Reference	0.635
	ILC	0.818 (0.665-1.005)		1.501 (0.734-3.067)	
	IDC+ILC	1.178 (0.947-1.467)		1.659 (0.772-3.561)	
	Other	0.390 (0.271-0.553)		1.553 (0.696-3.464)	

### Construction and validation of the prediction nomogram

**Figure 1:**
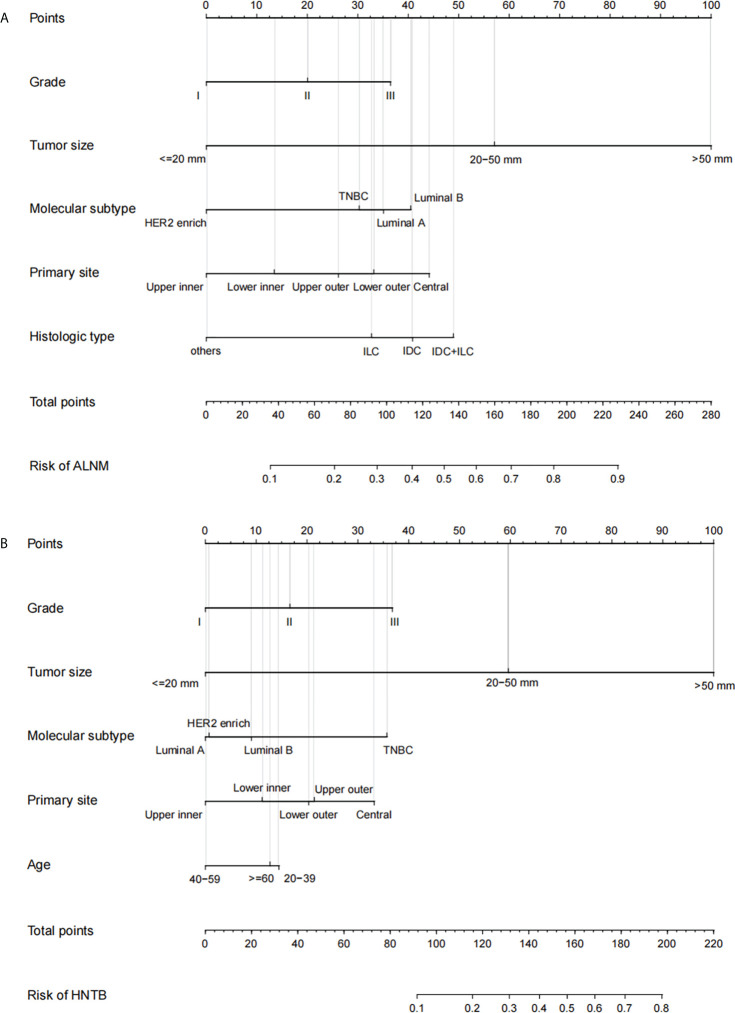
The nomograms of prediction model in breast cancer patients (cN0) Note: **(A)** the prediction model 1 of axillary lymph node metastasis (ALNM, LN− vs. LN+); **(B)** the prediction model 2 of high nodal tumor burden [HNTB, LN− and LN+(1–2) vs. LN+(>2)]. LN−, disease-free axillae; LN+: any nodal metastasis; LN+(1–2), 1 or 2 nodal metastasis; LN+(>2), more than 2 nodal metastasis; Grade I, well-differentiated, Grade II, moderately differentiated, and Grade III, poorly differentiated; TNBC, triple-negative breast cancer; IDC, invasive ductal carcinoma; ILC, invasive lobular carcinoma; others: mucinous carcinoma, medullary carcinoma, tubular carcinoma, and metaplastic carcinoma.

**Figure 2:**
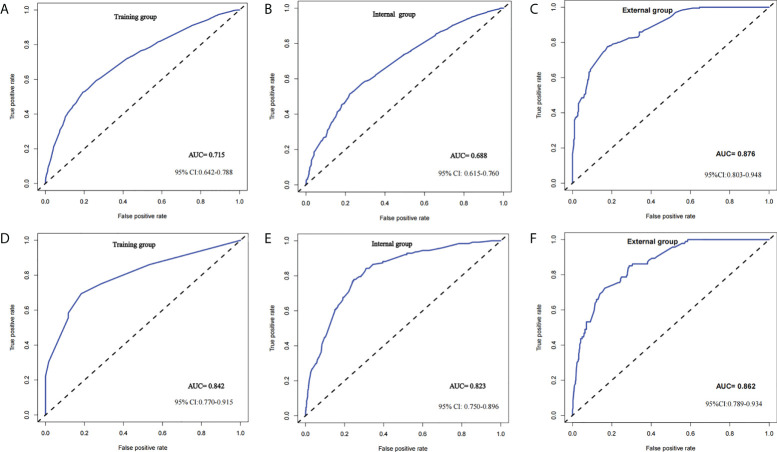
ROC curve of predictive model in breast cancer (cN0) Note: **(A–C)** ROC curve of predicting ALNM (LN− vs. LN+) in the training group, internal group and external group; **(D–F)** ROC curve of predicting HNTB [LN− and LN+ (1–2) vs. LN+ (>2)] in the training group, internal group and external group.

**Figure 3:**
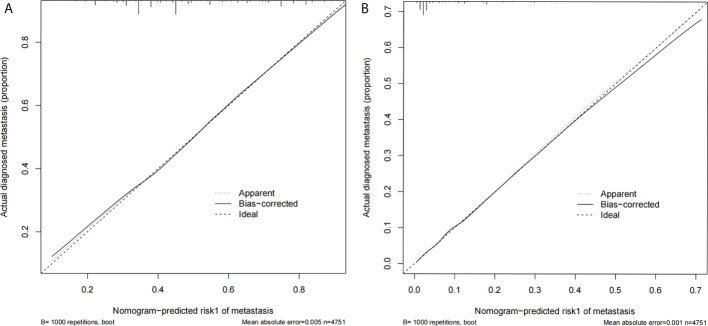
**(A)** The calibration curve for prediction model 1 of ALNM (LN− vs. LN+); **(B)** the calibration curve for prediction model 2 of HNTB [LN− and LN+ (1–2) vs. LN+ (>2)].

Two nomograms (**Figure 1**) based on significant and independent predictors determined by multivariate logistic regression analysis were established to predict the ALNM and HNTB of breast cancer patients (cN0). By adding the scores of the corresponding predictors in the respective nomograms, the probability of ALNM and HNTB in a specific patient can be predicted. The effectiveness of the nomograms for predicting ALN status was further evaluated by using ROC curves for the training and validation groups (**Figure 2**). In the training group, the AUC of ALNM was 0.715 (95% CI: 0.642-0.788), which was similar to the AUCs observed in the internal validation group (0.688, 95% CI: 0.615-0.760) and in the external validation groups (0.876, 95% CI: 0.803-0.948). In another training group, the AUC of HNTB was 0.842 (95% CI: 0.770-0.915), and the AUCs were 0.823 (95% CI: 0.750-0.896) and 0.862 (95% CI: 0.789-0.934) in the corresponding internal validation and external validation groups, respectively. To test the performance of the nomograms, 1,000 bootstrap re-samplings were used for internal verification through calibration charts in each training group (**Figure 3**). The two calibration curves indicated good calibration effects of the corresponding nomograms.

## Discussion

At present, breast cancer is one of the leading diseases that threatens the health of women (2). Although breast cancer relies on comprehensive treatment, including but not limited to surgery, adjuvant chemotherapy, radiotherapy and even immunotherapy, to delay disease progression and improve prognosis, accurate preoperative N staging of breast cancer is of great importance for the selection of an appropriate surgical approach and subsequent individualized comprehensive treatment (26). In particular, the ACOSOG Z0011 trial indicated that a therapeutic strategy for axillary surgery based on the number of positive ALNs became particularly important. Therefore, we established prediction models to accurately predict the status of ALNs.

In this study, the training group was divided into patients without ALNM, 1-2 positive ALNs, and >2 positive ALNs. Our study included 11 clinicopathological features as potential predictors for ALN status in breast cancer. Her-2 status and laterality were associated with ALNM according to the univariate analysis. In contrast, they were not identified as independent factors through multivariate logistic analyses. Finally, by univariate analysis and multivariate logistic regression analysis, we found that tumour size, primary site, molecular subtype and grade were independent predictors of both ALNM and HNTB, which was consistent with the results of previous studies (27–29).

Tumour size was found to be an indisputable independent predictor for ALNM, which was confirmed in many previous studies (30–32). In addition, the consensus was that the larger the tumour is, the greater the likelihood of metastasis to the ALNs. It was concluded that the site of the primary tumour was particularly important for predicting ALN status in this study. In particular, patients with primary tumours located in the central breast are more likely to metastasize to the ALN, which was also consistent with the view of Chen et al. (29, 33). As for histological grade, it is still controversial. Currently, some studies have shown that there is no correlation between grade and ALNM (34, 35). However, in addition to our study, other studies also found that grade II and III cancers were more prone to ALN metastasis than grade I cancers (36, 37).

Moreover, in our study, histologic type and age were independent predictors of ALNM and HNTB, respectively. Tan et al. also showed that non-ductal or lobular histological type (such as mucinous, medullary, tubular or metaplastic carcinoma) compared to invasive ductal or lobular type was associated with a lower risk of ALNM (38, 39). A study showed that compared with older patients, young patients are more likely to have regional lymph node and distant metastasis (40). Other studies have considered age as an independent predictor of ALNM (27–29, 41). We found that HNTB was more likely to occur in young women (age<40).

Finally, based on the above predictors, two different graphical nomograms were developed. The AUCs of the nomograms in ROC curve analysis were 0.715 and 0.842, respectively; internal and external validation showed good discrimination in the prediction of the three ALN statuses. The nomograms can be used to provide a basis for appropriate personalized ALN treatment strategies.

Our study aimed to identify predictors through relevant clinicopathological indicators with a large population size and establish prediction models to reduce unnecessary invasiveness by preoperative prediction. First, our study suggested that SLNB may be avoided in patients with a minimal risk of ALNM if conditions allow (T1-2, cN0, the planned breast-conserving surgery and radiotherapy). Although SLNB is a minimally invasive and well-tolerated surgery, serious allergic reactions and radiation safety caused by tracers of SLNB have been reported (42, 43). Moreover, sentinel lymph node metastasis only exists in 30-35% of patients, and SLNB seems to benefit only a small number of patients (42). More clinical trials are investigating whether SLNB can safely be omitted in breast cancer patients (cN0), such as the SOUND and BOOG 13-08 trials, the results of which are expected in the next few years (44, 45). Second, our study suggested that ALND should be avoided in patients with 1-2 positive SLNs, which is consistent with the prediction result (LNTB). Currently, NCCN guidelines also recommend that for patients with T1 or T2 tumours and 1 to 2 positive SLNs treated with lumpectomy but no preoperative systemic therapy, further axillary surgery could be omitted (26). Finally, we suggested that for predictive HNTB patients (> 2 positive ALNs), who need appropriate neoadjuvant therapy before surgery.

There are several highlights in the study (1): To the best of our knowledge, this is the first internally and externally validated nomogram for accurately stratified prediction of ALN status in female breast cancer patients based on clinicopathological features. Most of the previous studies can only predict ALN status (such as metastasis or no metastasis) or have not been validated (especially external validation), which decreases study credibility (27, 28, 46) (2). Our nomograms can be used extensively with common clinical clinicopathological features, such as tumour size, primary site, grade, age, and molecular subtype, which can be obtained preoperatively (3). This study included a large population size and was validated with external data, which indicated its reliability.

However, this study still has some limitations (1): As a retrospective study, selection or information bias was unavoidable (2). We were unable to obtain additional information from the SEER database, including ultrasound features, oncogenes and more pathological features. If the above information was included, the sensitivity and specificity of the current nomograms would be enhanced (3). The external validation cohort was from a single-center, the number of cases in external validation groups is still significantly less than that in training group (4). Previous study showed that the non-Hispanic black women have a worse prognosis with higher mortality rate (1, 47), it would be a potential predictor for axillary lymph node metastasis. However, the detailed race information wasn’t provided in the SEER database.

## Conclusion

In conclusion, we established two nomograms based on common clinical pathology features, including tumour size, primary site, molecular subtype, grade, histologic type and age, to accurately predict the risk of preoperative ALNM and HNTB in breast cancer patients (cN0). The quantitative risk assessment and prediction provided by the nomograms can help doctors avoid additional invasive treatment and provide a new individual strategy for the management of breast cancer patients (cN0).

## Data availability statement

The original contributions presented in the study are included in the article/supplementary material. Further inquiries can be directed to the corresponding author.

## Ethics statement

The studies involving human participants were reviewed and approved by Ethics Committee of the Second Affiliated Hospital of Chongqing Medical University. Written informed consent for participation was not required for this study in accordance with the national legislation and the institutional requirements.

## Author contributions

LY and XG designed the study and wrote the main manuscript text. XG and WL provided the study materials or patients and analyzed all data. LH and LY revised the manuscript. All authors read and approved the final manuscript.

## Funding

This study was supported by Chongqing Natural Science Foundation (grant no. cstc2020jcyj-msxmX2011), Science and Technology Bureau of Yuzhong District, Chongqing (grant no. 20200145), and Kuanren Talents Program of the second affiliated hospital of Chongqing Medical University.

## Acknowledgments

We would like to thank SEER for providing open access to the database.

## Conflict of interest

The authors declare that the research was conducted in the absence of any commercial or financial relationships that could be construed as a potential conflict of interest.

## Publisher’s note

All claims expressed in this article are solely those of the authors and do not necessarily represent those of their affiliated organizations, or those of the publisher, the editors and the reviewers. Any product that may be evaluated in this article, or claim that may be made by its manufacturer, is not guaranteed or endorsed by the publisher.
